# The relationship between home-kindergarten-community collaboration and socioemotional competence among Chinese preschoolers: the chain mediating roles of principals’ transformational leadership and teachers’ socioemotional competence

**DOI:** 10.3389/fpsyg.2025.1647323

**Published:** 2025-09-12

**Authors:** Muxiang Sun, Zhiwen Feng, Liangyong Xiao

**Affiliations:** Faculty of Education, Yunnan Normal University, Kunming, China

**Keywords:** home-kindergarten-community collaboration, preschoolers’ socioemotional competence, principals’ transformational leadership, teachers’ socioemotional competence, chain mediation

## Abstract

**Objective:**

This study sought to investigate the impact of home-kindergarten-community collaboration on children’s socioemotional competence and its underlying mechanisms.

**Methods:**

To this end, an online questionnaire was administered to 4,780 kindergarten teachers in Guangdong and Yunnan Provinces. The survey solicited their perceptions regarding the current status of home-kindergarten-community collaboration and their assessments of the director’s transformational leadership. Additionally, the developmental levels of children’s and teachers’ socioemotional competence were assessed through teacher reports.

**Results:**

The findings showed substantial disparities in the development of children’s socioemotional competence based on gender, age, and geographical location. Additionally, notable variations existed among different kindergarten types in terms of home-community collaboration, directors’ transformational leadership, and children’s socioemotional competence. Furthermore, substantial positive correlations were identified between home-kindergarten-community collaboration and children’s socioemotional competence and between directors’ transformational leadership and teachers’ socioemotional competence. Specifically, home-kindergarten-community collaboration indirectly promotes the development of children’s socioemotional competence through principals’ transformational leadership and teachers’ socioemotional competence.

**Conclusion:**

Kindergarten principals and teachers should fortify their central roles to foster collaboration between the home and society. They must meticulously create nurturing physical environments and establish comprehensive emotional support systems to ensure the optimal development of children’s socioemotional competence.

## 1 Introduction

The socioemotional competence of preschoolers serves as a critical cornerstone for lifelong well-being, academic success, and positive social integration (Denham, 2006; Jones et al., 2016). Recognizing its profound significance, researchers and practitioners are increasingly emphasizing the pivotal role of supportive ecosystems in nurturing children’s socioemotional competence development. Within this context, home-kindergarten-community collaboration has emerged as a fundamental ecological system influencing young children’s development (Bronfenbrenner, 1979). Extant research robustly underscores the individual importance of home-kindergarten partnerships and community involvement for children’s positive outcomes, including enhanced socioemotional competence (e.g., Fantuzzo et al., 2000; Sheridan et al., 2019). Similarly, the influence of kindergarten principals’ transformational leadership—characterized by inspiring vision, individualized support, and intellectual stimulation—in fostering positive organizational climates conducive to teacher well-being and potentially, child development is gaining empirical support (e.g., Pitkäniemi et al., 2025; Hallinger and Heck, 2010). Concurrently, teachers’ own socioemotional competence is recognized as a vital factor influencing classroom climate, teacher-child interactions, and consequently, children’s socioemotional competence (Jennings and Greenberg, 2009).

Despite the significance of socioemotional competence, considerable gaps persist in our understanding. Although previous studies have explored the direct links between home-kindergarten-community collaboration and children’s socioemotional competence and between principals’ transformational leadership and teacher outcomes, the integrated mechanisms through which HC collaboration indirectly influences children’s socioemotional competence remain inadequately understood. Crucially, the potential chain mediating roles of kindergarten principals’ transformational leadership and teachers’ socioemotional competence in transmitting the benefits of broader home-kindergarten-community collaboration to the child level constitute a critical but underexplored pathway (Sheridan et al., 2019). Specifically, there is insufficient empirical investigation examining how home-kindergarten-community collaboration promotes preschoolers’ socioemotional competence sequentially through enhancing principals’ transformational leadership and subsequently, teachers’ socioemotional competence, particularly within diverse Chinese contexts, which is the core research focus of this study. Understanding this specific chain of influence is paramount for designing targeted interventions that leverage the synergistic potential of these interconnected factors.

Furthermore, contemporary research calls for greater attention to the contextual factors shaping collaboration effectiveness and leadership practices in early childhood education (Douglass, 2019). The Chinese context, with its unique cultural emphasis on education, evolving family structures, and diverse kindergarten types (public, private, urban, and rural), presents a compelling setting to investigate these complex relationships (Jin and Ke, 2024). Recent studies have highlighted both the potential and challenges of fostering effective home-kindergarten-community collaboration in China (Jinqiu and Hai, 2024), underscoring the timeliness and relevance of examining the specific pathways through which such collaboration impacts children’s socioemotional competence outcomes.

Therefore, this study aims to address these critical gaps by explicitly investigating the chain mediating roles of principals’ transformational leadership and teachers’ socioemotional competence in the relationship between home-kindergarten-community collaboration and preschoolers’ socioemotional competence. Drawing on a large-scale sample of kindergarten teachers from diverse regions in China (Guangdong and Yunnan provinces), we seek to provide nuanced empirical evidence on how the collaborative efforts in homes, kindergartens, and communities translate, via the leadership of principals and the socioemotional capacities of teachers, into tangible benefits for young children’s social and emotional development. This investigation not only contributes to refining the theoretical models of ecological influence in early childhood but also offers actionable insights for strengthening collaborative practices and leadership development within the Chinese early childhood education system and potentially beyond.

## 2 Literature review and research hypotheses

### 2.1 Theoretical foundations

The ecological systems theory emphasizes that individual development is nested in the interactions of multi-level systems: the microsystem (family, kindergarten), mesosystem (interactions between systems), exosystem (social organizations), and macrosystem. As the core carrier of the mesosystem, collaboration among families, kindergartens, and the community fosters a consistent and continuous growth environment for young children through the in-depth integration of educational goals, resources, and methods (Chiliang and Maoqing, 2022). The “social interaction–individual internalization” trajectory posits that a consistent parenting model can enhance young children’s awareness of behavioral norms. The integration of resources has been demonstrated to promote various social interaction scenarios, and the establishment of a value consensus has been shown to enhance the emotional support experiences of young children. Principals’ transformational leadership, characterized by the transmission of vision, personalized care, and intellectual stimulation, has been demonstrated to have a significant impact on the organizational culture of kindergartens. Moreover, such leadership has played a pivotal role in establishing a collaborative mechanism of “family–kindergarten–community.”

Furthermore, ecological systems theory elucidates that principals can significantly enhance teachers’ social and emotional capabilities through the demonstration effect and organizational empowerment. This professional development is reflected not only in the enhancement of teachers’ self-efficacy but also in the optimization of emotional support strategies in teaching practice (Nursanti et al., 2025). Teachers with high levels of social–emotional skills are better able to identify young children’s developmental needs. This finding aligns with the effective intervention principle of Bronfenbrenner’s “proximal process” theory. The mediating role of the quality of teacher-child interaction has been identified as a pivotal factor in this developmental process (Li et al., 2025).

The chain conduction mechanism between principals’ transformational leadership and teachers’ socioemotional competence reflects cross-system interactions among elements at all levels of the ecosystem. According to conservation of resources theory, transformational leadership provides teachers with two types of resources: psychological capital (e.g., professional identity) and professional capital (e.g., collaborative educational skills). This resource gain enables teachers to participate more actively in collaborative practices with their families, kindergartens, and communities. Meanwhile, transformative learning theory suggests that transformational leadership can foster the cognitive reconstruction and practical transformation of teachers’ socioemotional competence by encouraging critical reflection. This professional growth is evident in teachers’ increased sensitivity to children’s emotional needs and optimized communication and coordination strategies with parents and the community. Thus, an ecological cycle of “leadership empowerment–teacher development–children’s growth” is formed. This multilevel linkage mechanism verifies the core proposition of developmental systems theory that individual development is the product of dynamic interactions within nested systems.

### 2.2 Research hypotheses

#### 2.2.1 The role of home-kindergarten-community collaboration in improving preschoolers’ socioemotional competence

Home-kindergarten-community collaboration refers to a joint-effort education model formed through cooperation between families, kindergartens, and community stakeholders. Collectively, these elements constitute a micro-ecosystem critical for children’s development (McWhirter et al., 2023; Xiaoping and Guobin, 2024). International frameworks, such as the Collaborative for Academic, Social, and Emotional Learning (CASEL) model, emphasize that effective social and emotional learning (SEL) requires synergistic engagement across these contexts in which community resources reinforce school and family efforts in nurturing socioemotional skills. With the implementation of policies such as China’s Guidelines for Kindergarten Education and the Law on Promoting Family Education, research on tripartite collaboration has deepened. Notably, empirical studies from Western contexts have demonstrated that structured home-school-community programs significantly enhance preschoolers’ socioemotional outcomes. For example, Sheridan et al. (2019) conducted a meta-analysis of 40 interventions, revealing that family engagement boosted children’s social competence and reduced behavioral problems by 27% through shared decision-making and resource integration. Similarly, Bierman and Sheridan (2021) tracked a multiyear SEL program in the U.S., finding that parental involvement mediated 45% of gains in emotion regulation and prosocial behavior, underscoring the “overlapping spheres of influence” theory.

Current research on collaboration mechanisms remains predominantly theoretical. Theoretical (Li et al., 2020; Linlin et al., 2023; Ramos et al., 2025; Wang and Yuan, 2024; Wang et al., 2024; Xu et al., 2024) and empirical studies on PSC span:; the influence of factors such as family cultural capital (Lu and Jiangying, 2023); maternal emotional warmth (Yichu and Jiahao, 2025); types of teacher-student relationships (Li and Bai, 2024); children’s executive functions (Fung and Chung, 2023); parent–child dysfunction (Liu et al., 2024), effects of parental relationships on children’s socioemotional abilities (Tang et al., 2023), and cultivation strategies (Hosokawa et al., 2024; Wenbin et al., 2025). However, rigorous validation of the impact of home-kindergarten-community mechanisms’ on socioemotional skills is scarce. Ahmed et al. (2022) linked community partnership sustainability to 30% higher teacher efficacy in SEL delivery. Given the global evidence on home-school communication benefits (e.g.,), we posit a significant positive association between this collaboration and socioemotional competence.

Therefore, this study proposes the following:

Hypothesis 1: Home-kindergarten-community collaboration positively promotes preschoolers’ socioemotional competence.

#### 2.2.2 The mediating role of kindergarten principals’ transformational leadership

The leadership of kindergarten principals refers to their capacity to exert influence over all teaching and administrative staff within the kindergarten, as well as to engage key stakeholders, including children and parents, within a specific context. This involves guiding these individuals in establishing and achieving the organizational goals of kindergartens, thereby promoting comprehensive development (Linfang and Haimin, 2015). Kindergarten principals exhibit various leadership types, including value-based, educational, curriculum, and transformational leadership (Meng, 2014; Lijuan, 2020; Siyu, 2020; Jiao et al., 2021; Fang and Hao, 2022; Dongxia, 2024). Among these, transformational leadership is specifically characterized by a range of leadership behaviors, including fostering a cooperative kindergarten culture, establishing clear directions, reorganizing the institutional structure, enhancing teaching methodologies, and promoting teachers’ professional development. These actions can inspire teachers to pursue higher-level needs, reinforce their intrinsic motivation, and shape their beliefs and values. Consequently, this leadership style enables teachers to surpass their job performance expectations, collaboratively manage challenges, and realize superior levels of achievement and effectiveness (Dongxia, 2024). Similar patterns have been observed worldwide. For instance, Pitkäniemi et al. (2025) documented how Finnish principals’ transformational practices directly increased teacher autonomy and pedagogical innovation, while Wang et al. (2025) demonstrated the mediating role of principals’ transformational leadership in translating community partnerships into teacher well-being in Beijing, China. Overall, principals’ transformational leadership is reflected in four core areas: setting a clear direction, fostering personnel development, enhancing teaching quality, and reorganizing the institutional structure. Studies have shown that transformational leadership by kindergarten principals positively correlates with outcomes such as online support for children and teacher work engagement (Siyu, 2020; Dongxia, 2024). Additionally, the organizational climate within kindergartens is crucial in moderating the effectiveness of principals’ transformational leadership (Meng, 2014; Fang and Hao, 2022). This aligns with Douglass (2019) OECD analysis emphasizing the pivotal role of leadership in mediating home-community collaborations for sustainable child development.

Based on these insights, this study proposes the following:

Hypothesis 2: Principals’ transformational leadership mediates the relationship between home-kindergarten-community collaboration and preschoolers’ socioemotional competence.

#### 2.2.3 The mediating role of preschool teachers’ socioemotional competence

Teachers’ socioemotional competence involves the ability to recognize and manage their own emotions as well as those of others, actively regulate negative emotions, understand the psychological states of others, and establish harmonious interpersonal relationships with children, parents, and colleagues in educational settings and collective life situations. Additionally, it encompasses the capacity to courageously face difficulties, effectively manage stress, reasonably address challenges in both professional and personal contexts, and exhibit behaviors and rational judgments that align with situational demands across various scenarios, including societal, familial, community, and kindergarten environments (Yan, 2023). This aligns with Jennings and Greenberg's (2009) prosocial classroom model, which positions teachers’ socioemotional competence as the foundation for creating supportive learning ecosystems. The core components of this competence include self-awareness, self-regulation, social awareness, interpersonal communication skills, and responsible decision-making. As the socioemotional competence of preschool teachers originates from general socioemotional competence but specifically emphasizes interactions within children’s groups, the academic community has conducted relevant research in this area, such as developing theoretical models and designing and piloting situational tests (Lin, 2022; Rong, 2022). Research indicates that teachers’ socioemotional competence is a critical factor in predicting the social and emotional development of young children. Empirical evidence from Partee et al. (2025) confirms that the emotional regulation skills of preschool teachers mediate the association between program quality and children’s empathy growth. Enhancing these competencies among preschool teachers requires a multifaceted approach that encompasses curriculum-based learning, kindergarten support, family influence, and the creation of an enriching kindergarten environment. International interventions, such as Singh’s (2024) mindfulness-based program for Head Start teachers, demonstrate that targeted SEL training boosts teachers’ emotional support practices, subsequently increasing children’s prosocial behaviors.

Based on this understanding, this study proposes the following:

Hypothesis 3: Teachers’ socioemotional competence mediates the relationship between home-kindergarten-community collaboration and children’s socioemotional competence.

#### 2.2.4 The chain-mediating role of principals’ transformational leadership and teachers’ socioemotional competence

Multiple studies have consistently shown that kindergarten principals’ leadership exerts a significant influence on various dimensions of kindergarten teachers’ professional development. From the perspective of work engagement ecosystem theory, kindergartens, families, and communities jointly constitute a complex ecosystem that supports children’s holistic development. This ecological framework aligns with Hayes (2018) serial mediation paradigm, which emphasizes sequential pathways in organizational contexts. Within this overarching system, the kindergarten is further divided into several interrelated subsystems: management, environmental, and curriculum and instruction systems. Collectively, these subsystems form a micro-ecological framework that underpins children’s learning and developmental processes. Notably, the collaboration between homes, kindergartens, and communities is pivotal in nurturing preschoolers’ socioemotional competence (Sun et al., 2025). Empirical evidence from Wang et al. (2025) confirms that principal-teacher chains mediate external collaboration effects on child outcomes in diverse cultural settings. This collaborative process is primarily driven by the complementary roles of principals and teachers, with teachers’ behaviors and competencies being significantly shaped by the leadership style adopted by the principal.

Based on the aforementioned, this study proposes the following:

Hypothesis 4: Principals’ transformational leadership and teachers’ socioemotional competence play a chained mediating role in the influence of home-kindergarten-community collaboration on preschoolers’ socioemotional competence.

In sum, this study aimed to explore how home-kindergarten-community collaboration affects the development of preschoolers’ socioemotional competence within the kindergarten context and to reveal the chain mediating effect between principals’ transformational leadership and teachers’ socioemotional competence, as well as its specific mechanism of action.

**Figure 1:**
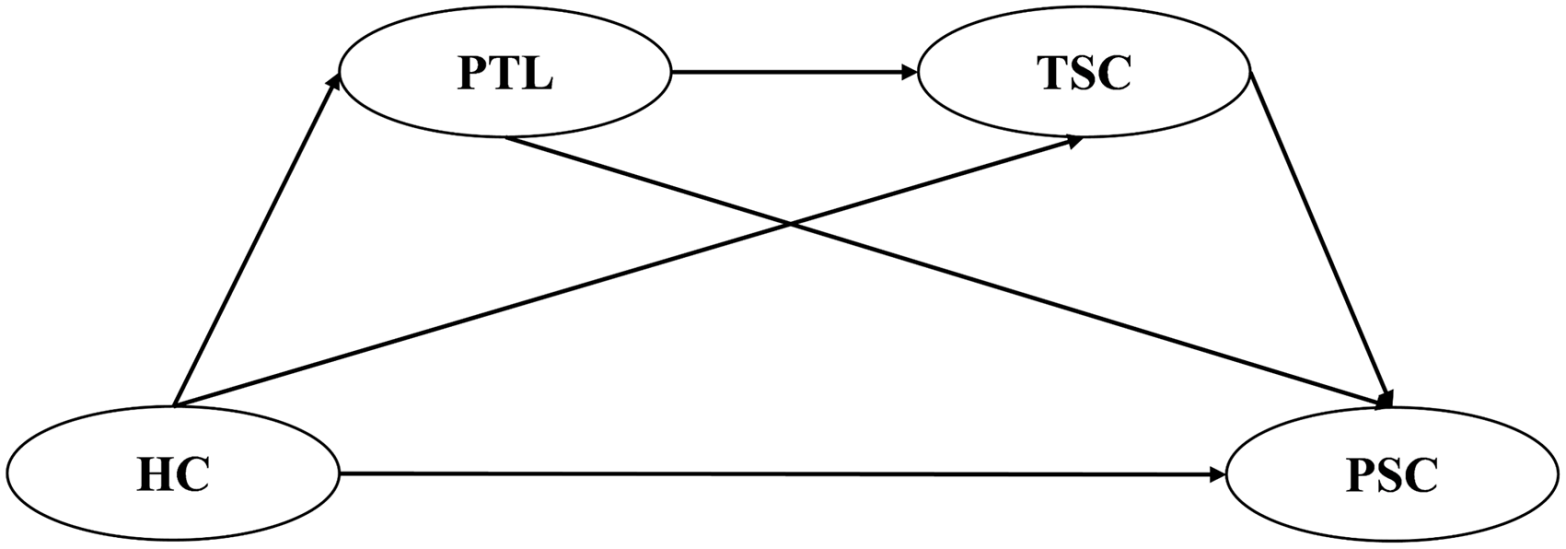
A hypothetical model of the home-kindergarten-community collaboration influence on preschoolers’ socioemotional competence. HC, home-kindergarten-community collaboration; PSC, preschooler’s socioemotional competence; PTL, principals’ transformational leadership; TSC, teachers’ socioemotional competence.

The hypothesized model of the study is illustrated in Figure 1.

## 3 Materials and methods

### 3.1 Participants

This study employed a convenience cluster sampling method to survey kindergarten teachers in Guangdong and Yunnan provinces in Eastern and Western China. A total of 5,635 questionnaire invitations were distributed. After rigorous review, 855 questionnaires were deemed invalid and excluded based on the following criteria: (a) completion time was too short (less than one-third of the median completion time); (b) exhibited patterns of careless responses (e.g., selecting the same option for multiple questions); and (c) failed to pass at least two embedded attention check questions. Consequently, 4,780 valid questionnaires were obtained, yielding an effective response rate of 84.83%. Teachers provided demographic information about the children in their class(es) whom they reported on, which may be subject to social desirability or recall biases. Table 1 presents the descriptive statistics of the subjects.

**Table 1:** Descriptive statistics of the subjects.

Category	Subcategory	Frequency	Percentage (%)
Child gender	Male	2,429	50.82
	Female	2,351	49.18
Child age	≤ 3 years	220	4.60
	3–4	1,286	26.90
	4–5	1,503	31.44
	5–6	1,471	30.77
	≥ 6 years	300	6.28
Kindergarten type	Public	3,368	70.46
	Private	1,412	29.54
Kindergarten area	Urban	3,184	66.61
	Town	818	17.11
	Rural	778	16.28
Total		4,780	100.0

### 3.2 Measures

#### 3.2.1 Home-kindergarten-community collaboration scale

The Home-Kindergarten-Community Collaboration Scale was derived from the “Toward Quality-Evaluation Criteria for the Quality of Kindergarten Education in China,” developed by the Preschool Education Research Association of China. This quality standard comprises five core quality domains, with the fifth domain being “Home-Kindergarten-Community Collaboration.” This domain includes four evaluation dimensions: overall management, home-kindergarten communication, home-kindergarten cooperation, and community co-construction, which are further subdivided into nine specific sub-items. A seven-point scoring system is employed, and the quality level of home-kindergarten-community collaboration is assigned four grades: “Inadequate (1),” “Qualified (3),” “Good (5),” and “Excellent (7).” Additionally, the overall internal consistency Cronbach’s *α* coefficient of the “Quality Standard” was 0.926, whereas the internal consistency α coefficients for each domain ranged from 0.55 to 0.934, indicating that the “Quality Standard” exhibits strong internal consistency at both the overall and domain levels, thereby ensuring the stability and reliability of the evaluation outcomes (Dezhi et al., 2021).

#### 3.2.2 Principals’ transformational leadership scale

The Principals’ Transformational Leadership Scale was derived from the “Chinese Transformational School Leadership Questionnaire” developed by Liu et al. (2024) and was adapted by Dongxia (2024) into the “Principals’ Transformational Leadership Scale,” which is specifically designed for kindergarten principals. The scale comprises four dimensions: developing personnel, improving teaching, restructuring the organization, and setting the direction. There are 22 questions on a 5-point scale (strongly disagree = 1, disagree = 2, neutral = 3, agree = 4, strongly agree = 5). The scale had good model data fit (CFI = 0.892, RMSEA = 0.079, SRM*R* = 0.047), and the four subscales showed good internal agreement (Cronbach’s = 0.982) (Dongxia, C., 2024).

#### 3.2.3 Preschool teachers’ socioemotional competence scale

The Preschool Teachers’ Socioemotional Competence Scale (Ma, 2023) was psychometrically adapted from Wang (2021) Social and Emotional Competence Questionnaire for Primary and Secondary School Teachers to suit preschool educational contexts. This questionnaire comprises five dimensions, namely self-awareness, self-regulation, social awareness, interpersonal communication, and responsible decision-making, all of which are contextualized for kindergarten teachers. The scale includes 38 items and uses a 5-point rating scale (1 = strongly disagree, 2 = disagree, 3 = neutral, 4 = agree, 5 = strongly agree). Preliminary testing indicated that the scale exhibits excellent reliability (Cronbach’s *α* = 0.962) and validity (KMO value = 0.938) and has been officially adopted for use in relevant research (Yan, 2023).

#### 3.2.4 Children’s socioemotional competence scale

The Children’s Socioemotional Competence Scale was adopted from the Chinese scale developed by Li et al. (2020) at the Education University of Hong Kong in 2020. Li et al. (2020) explored the structure of socioemotional competence in kindergarten children in Hong Kong and developed and verified a socioemotional competence assessment tool for children reflective of their Chinese cultural background. After multiple rounds of confirmatory factor analysis, the final scale comprised four core dimensions: cognitive control, emotional expressivity, empathy and prosocial behaviors, and emotional regulation. It included 30 questions on a 5-point scale (strongly disagree = 1, disagree = 2, neutral = 3, agree = 4, strongly agree = 5). The scale had good model data fit (CFI = 0.91, RMSEA = 0.055, SRM*R* = 0.053), and the four subscales showed good internal agreement (Cronbach’s α ranged from 0.83 to 0.92) (Li et al., 2020).

### 3.3 Process

Ethical approval was obtained from the Ethics Committee of the First Author’s University. The questionnaire survey was administered through the Wenjuanxing platform (www.wjx.cn) over a six-week period from mid-November to late December 2024. Questionnaire links were distributed to kindergarten principals/administrators in selected cities across Eastern (Foshan, Guangzhou, Shenzhen, and Chaozhou in Guangdong Province) and Western regions (Pu′er in Yunnan Province) of China via collaboration with local education authorities. Principals were requested to forward the link to teachers within their kindergartens. Prior to accessing the questionnaire, all potential participants were presented with a detailed online informed consent form on the Wenjuanxing platform. This form outlined the study objectives, procedures, estimated time commitment, data confidentiality measures, voluntary nature of participation, and anonymity of responses. Participants were required to submit their consent electronically (‘Agree’) before proceeding. All responses were collected voluntarily and anonymously, strictly adhering to the principles of information confidentiality and participant anonymity.

### 3.4 Data analysis

We employed SPSS software (version 22.0) for data entry and statistical analysis. Specifically, the analyses encompassed common method bias (CMB) tests, descriptive statistics, independent samples t-tests, and one-way analysis of variance (ANOVA) to examine the variations in home-kindergarten-community collaboration, preschool teachers’ socioemotional competence, principals’ transformational leadership, and preschoolers’ socioemotional competence across factors such as gender, age, type of kindergarten, and geographical location. Additionally, AMOS software (version 26.0) was used to test the chain mediation model, further exploring the mechanism through which home-kindergarten-community collaboration influences preschoolers’ socioemotional competence. Given that all data were collected via questionnaires, including reverse-scored items, a potential CMB could arise. To mitigate this concern, we implemented a series of rigorous control measures based on recommendations from the relevant literature. An anonymous evaluation approach was adopted during data collection, and the scale items were randomly arranged. Furthermore, questionnaire instructions and scale structures were differentially designed and combined with a strictly standardized testing to minimize participants’ ability to guess the measurement purpose. Prior to data analysis, the Harman single-factor test was conducted to assess CMB. The results revealed the presence of 11 factors with eigenvalues greater than 1, and the variance explained by the first factor was 26.04%, which was below the critical threshold of 40%, indicating that no significant CMB existed in the dataset.

## 4 Results

### 4.1 Differences in the four dimensions by gender, age, type of kindergarten, and geographical location

This study utilized an independent samples t-test to investigate differences across four dimensions—home-kindergarten-community collaboration, preschool teachers’ socioemotional competence, principals’ transformational leadership, and preschoolers’ socioemotional competence—between public and private kindergartens. The findings (see Table 2) indicated significant variations in these dimensions based on kindergarten type. Specifically, public kindergartens scored significantly higher in home-kindergarten-community collaboration and principals’ transformational leadership than private kindergartens. By contrast, private kindergartens achieved significantly higher scores in preschool teachers’ socioemotional competence and preschoolers’ socioemotional competence than public kindergartens.

**Table 2:** Analysis of the differences in various variables in terms of gender, age, type of kindergarten, and geographical location.

Category	Subcategory	HC	TSC	PTL	PSC
Child gender	Male	4.56 ± 1.30	4.14 ± 0.77	4.23 ± 0.86	3.65 ± 0.57
	Female	4.52 ± 1.28	4.16 ± 0.78	4.25 ± 0.85	3.71 ± 0.55
T		0.847	0.411	−0.546	−3.449***
Kindergarten type	Public	4.58 ± 1.29	4.13 ± 0.77	4.28 ± 0.84	3.62 ± 0.51
	Private	4.45 ± 1.31	4.20 ± 0.79	4.12 ± 0.87	3.81 ± 0.64
T		3.151**	−3.097**	6.11***	−10.781***
Child age (Year)	<3	4.23 ± 1.46	4.00 ± 0.80	3.99 ± 0.80	3.50 ± 0.54
	3–4	4.53 ± 1.28	4.09 ± 0.78	4.18 ± 0.87	3.62 ± 0.55
	4–5	4.58 ± 1.25	4.14 ± 0.79	4.29 ± 0.81	3.69 ± 0.55
	5–6	4.53 ± 1.34	4.23 ± 0.78	4.27 ± 0.87	3.72 ± 0.56
	6–7	4.65 ± 1.18	4.23 ± 0.53	4.28 ± 0.78	3.68 ± 0.56
F		4.182**	8.755***	8.382***	18.012***
Kindergarten area	City	4.56 ± 1.30	4.17 ± 0.85	4.23 ± 0.86	3.63 ± 0.56
	Town	4.64 ± 1.17	4.16 ± 0.55	4.33 ± 0.79	3.85 ± 0.53
	Rural	4.36 ± 1.38	4.08 ± 0.64	4.17 ± 0.87	3.71 ± 0.56
F		10.384***	4.358*	7.195***	52.585***

*p < 0.05; **p < 0.01; ***p < 0.001.

Additionally, this study utilized a one-way ANOVA to examine the differences in preschoolers’ socioemotional competence indicators across different age groups. The results demonstrated significant variations in preschoolers’ socioemotional competence at different ages (Table 2). *Post hoc* multiple comparisons further revealed that besides the no significant difference in preschoolers’ socioemotional competence between the 4–5 and 5–6 year-old groups (*p* = 0.110), statistically significant differences were observed among the remaining groups (*p* < 0.001). A subsequent trend analysis indicated that as children progressed from the 1–3 to the 5–6 year-old group, their mean scores for preschoolers’ socioemotional competence exhibited a consistent upward trend with increasing age.

Finally, a one-way ANOVA was used to explore the differences in the indicators for four dimensions across different kindergarten locations. The results are summarized in Table 2. The location of kindergartens had a significant impact on all aforementioned four indicators. Specifically, rural and township kindergartens had significantly higher scores in terms of home-kindergarten-community collaboration than urban kindergartens (*p* < 0.001).

### 4.2 Correlation analysis of the four variables

Because all variables met assumptions of continuous measurement and normality, bivariate relationships were analyzed using Pearson’s correlation. Correlations among the variables are presented in Table 3. Specifically, home-kindergarten-community collaboration demonstrated a significant and positive correlation with principals’ transformational leadership (*r* = 0.438, *p* < 0.01), preschool teachers’ socioemotional competence (*r* = 0.397, *p* < 0.01), and preschoolers’ socioemotional competence (*r* = 0.260, *p* < 0.01). This suggests that stronger collaboration between homes, kindergartens, and communities is associated with enhanced principal transformational leadership, greater teacher socioemotional competence, and improved preschooler socioemotional competence. Furthermore, principals’ transformational leadership is significantly and positively correlated with preschool teachers’ socioemotional competence (*r* = 0.550, *p* < 0.01) and preschoolers’ socioemotional competence (*r* = 0.405, *p* < 0.01). These results indicated that principals who demonstrate transformational leadership behaviors, such as articulating a shared vision, fostering professional development, and offering individualized support, are more likely to enhance the socioemotional competencies of both teachers and children in early childhood education contexts. Moreover, a significant positive correlation exists between preschool teachers and preschoolers’ socioemotional competence (*r* = 0.459, *p* < 0.01). Finally, a significant positive correlation was observed between preschool teachers’ socioemotional competence and preschoolers’ socioemotional competence (*r* = 0.459, *p* < 0.01).

**Table 3:** Correlations among variables.

Category	M ± SD	1	2	3	4
1 HC	4.54 ± 1.29	1	-	-	-
2 PTL	4.24 ± 0.85	0.438**	1	-	-
3 TSC	4.15 ± 0.78	0.397**	0.550**	1	-
4 PSC	3.68 ± 0.56	0.260**	0.405**	0.459**	1

**p < 0.01.

These results are consistent with the theoretical expectations, indicating that teachers capable of effectively understanding, regulating, and modeling social–emotional skills are more likely to foster the development of these competencies in children. Collectively, these findings provide preliminary evidence for potential synergistic relationships among home-kindergarten-community collaboration, leadership practices, and social–emotional development across multiple levels within the early childhood education ecosystem.

### 4.3 Chain mediation effect between variables

**Figure 2:**
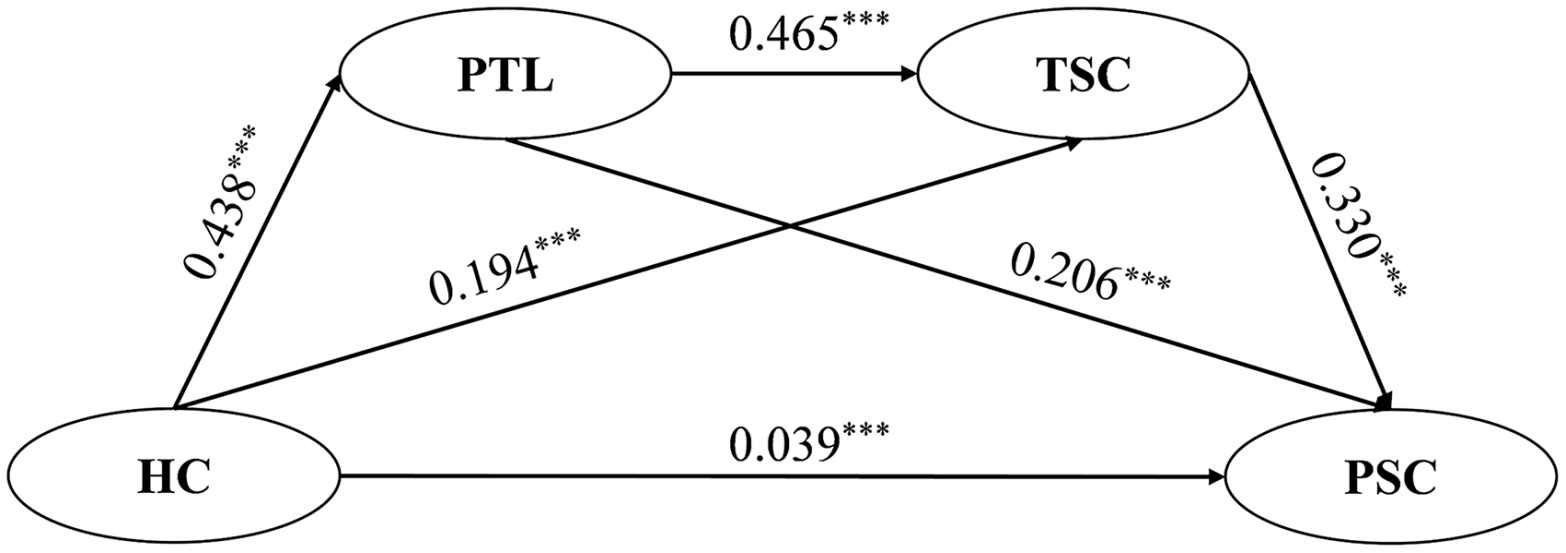
The chain mediation model illustrating the path relationships among variables.

This study examined the chain mediation by constructing a structural equation model. The independent variable was home-kindergarten-community collaboration, the dependent variable was preschoolers’ socioemotional competence, and the mediator variables were principals’ transformational leadership and teachers’ socioemotional competence. The chain mediation model results and path relationships are depicted in Figure 2, with GFI = 0.934, RMSEA = 0.063, RM*R* = 0.019, CFI = 0.959, NFI = 0.957, NNFI = 0.941, TLI = 0.941, and SRM*R* = 0.030. The chain mediation model demonstrated an adequate fit.

As shown in Figure 2, home-kindergarten-community collaboration significantly and positively predicts preschooler socioemotional competence (*β* = 0.039, *p* < 0.001), principal transformational leadership (*β* = 0.438, *p* < 0.001), and teacher socioemotional competence (*β* = 0.194, *p* < 0.001), thereby supporting Hypothesis 1. Moreover, principal transformational leadership significantly and positively predicts teacher (*β* = 0.465, *p* < 0.001) and preschooler socioemotional competence (*β* = 0.206, *p* < 0.001). Additionally, the teacher socioemotional competence is a significant positive predictor of preschooler socioemotional competence (*β* = 0.330, *p* < 0.001).

In this study, the bias-corrected bootstrap method was utilized to evaluate the significance of both the direct and chain mediating effects between home-kindergarten-community collaboration and preschooler socioemotional competence. The resampling procedure was performed 5,000 times. As shown in Table 4, the direct promoting effect of home-kindergarten-community collaboration on preschooler socioemotional competence was 0.039, accounting for 15% of the total effect. Importantly, the 95% confidence interval of the total mediating effect did not include zero, indicating a significant mediating effect accounting for 85% of the overall impact. The 95% confidence interval of the indirect effect path “home-kindergarten-community collaboration → principals’ transformational leadership of kindergarten principals → preschoolers’ socioemotional competence “did not include zero, indicating a statistically significant mediating effect. This mediating path contributed 0.090 to the enhancement of preschoolers’ socioemotional competence, accounting for 40.72% of the total indirect effects. Therefore, Hypothesis 2 was supported. Similarly, the 95% confidence interval of the indirect effect path “home-kindergarten-community collaboration → teachers’ socioemotional competence → preschoolers’ socioemotional competence” did not include zero, confirming a significant mediating effect. This path contributed 0.064 to the improvement in young children’s social–emotional skills, representing 28.96% of the total indirect effects. Hence, Hypothesis 3 was supported. Furthermore, the 95% confidence interval of the indirect effect path “home-kindergarten-community collaboration → principals’ transformational leadership → teachers’ socioemotional competence → preschoolers’ socioemotional competence” did not include zero, providing additional evidence of a significant mediating effect. This path contributed 0.067 to the enhancement of preschoolers’ socioemotional competence, accounting for 30.32% of the total indirect effects. Thus, Hypothesis 4 was supported.

**Table 4:** Analysis of mediation effects.

Pathway	Mediation effect value	95%Cis	Standard error	Proportion of effect
Total effect	0.260	(0.233,0.288)	0.014	100%
Direct effect	0.039	(0.011,0.067)	0.014	15%
Total mediation effect	0.221	(0.204,0.239)	0.009	85%
HC→PTL→PSC	0.090	(0.076,0.105)	0.007	40.72%
HC→TSC→PSC	0.064	(0.054,0.074)	0.005	28.96%
HC→PTL→TSC→PSC	0.067	(0.059,0.076)	0.004	30.32%

Bootstrap size = 5,000. CI: confidence interval.

## 5 Discussion

This study identified significant variations in preschoolers’ socioemotional competence across gender and age groups. Furthermore, it showed that the type and geographical location of kindergartens significantly influence home-kindergarten-community collaboration, principals’ transformational leadership, and teachers’ socioemotional competence. These factors collectively exert both direct and indirect effects on the development of preschoolers’ socioemotional competence. Specifically, effective home-kindergarten-community collaboration not only directly enhances preschoolers’ socioemotional competence but also indirectly promotes their overall social and emotional development by supporting principals’ transformational leadership and reinforcing teachers’ socioemotional competence.

### 5.1 Variable performance characteristics by age, gender, and location

Significant disparities were identified in the development of preschoolers’ socioemotional competence across gender and age groups. Specifically, girls consistently demonstrated higher proficiency in socioemotional competence than boys. This may be attributed to the gender-related advantages that girls exhibit in emotional perception, empathy, and prosocial behaviors, which are often reinforced by societal norms and expectations. Additionally, within the 3–6 year-old age range, older children tended to show more advanced socioemotional competence, aligning with established patterns of cognitive and social development during early childhood. However, children aged 6–7 years exhibited a decline in socioemotional competence levels compared to the 5–6 year-old group. This regression might have resulted from the impending transition from kindergarten to primary school, where insufficient support during this critical period could lead to temporary fluctuations in the developmental trajectory of preschoolers’ socioemotional competence.

Moreover, kindergartens of different types and geographical locations exhibited significant variations in home-kindergarten-community collaboration, preschoolers’ socioemotional competence, principals’ transformational leadership, and teachers’ socioemotional competence. This finding underscores the necessity of accounting for demographic variables when examining the influence of kindergarten-family-community collaboration on preschoolers’ socioemotional competence (Dezhi et al., 2021; Yichu and Jiahao, 2025). Specifically, public kindergartens tend to outperform private ones in terms of home-kindergarten-community collaboration, principals’ transformational leadership, and preschool teachers’ socioemotional competence. This could be attributed to the supportive policy framework that fosters stronger managers’ transformational leadership and teachers’ socioemotional competence in public kindergartens. In post-hoc multiple comparisons by geographical location, rural kindergartens situated at the urban–rural fringe demonstrated a relative advantage in kindergarten-family-community collaboration, principals’ transformational leadership, and teachers’ socioemotional competence. This phenomenon likely arises from the intricate interplay of factors such as economic conditions, family values, and power dynamics.

### 5.2 Home-kindergarten-community collaboration and preschoolers’ socioemotional competence

The results indicated that home-kindergarten-community collaboration is a significant and positive predictor of preschoolers’ socioemotional competence development. This finding further substantiates the efforts and contributions of the academic community in constructing and refining the theoretical framework and mechanisms for family-kindergarten-community collaboration (Fengmei, 2022; Juan et al., 2022; Hejie and Lei, 2023). Specifically, a more comprehensive home-kindergarten-community collaboration is associated with higher levels of socioemotional competence development in preschoolers. From the perspective of the nested system framework of ecological systems theory, children’s development is attributed to the dynamic interactions among the microsystem (family, kindergarten), mesosystem (home-kindergarten interaction), and exosystem (community resources). The positive predictive effect of family-kindergarten-community collaboration on preschoolers’ socioemotional competence underscores the added value of consistency across multiple systems for children’s development, thereby strongly supporting the core proposition of ecological systems theory: “development is a process of mutual adaptation between individuals and their environments.” Hence, this study not only provides robust empirical evidence for the effectiveness of the family-kindergarten-community collaborative education model in promoting children’s development but also delineates a clear pathway for optimizing this mechanism from the perspectives of kindergarten administrators and educators.

### 5.3 Mediating roles of the principal’s transformational leadership and teachers’ socioemotional competence

The findings reveal two mediating pathways between home-kindergarten-community collaboration and preschoolers’ socioemotional competence: “home-kindergarten-community collaboration → principals’ transformational leadership → preschoolers’ socioemotional competence” and “kindergarten-community collaboration→ teachers’ socioemotional competence → preschoolers’ socioemotional competence.” Following the ecosystem theory, the influence of the intermediate system—families, kindergartens, and communities—on early childhood development can be achieved through the organizational regulation of the outer system (e.g., the leadership of the director) and the transformation of the capacity of directly interacting subjects in the microsystem (e.g., teachers) (Bronfenbrenner, 1979). Specifically, as a key hub for transforming the synergistic environment into organizational effectiveness, directors’ transformational leadership systematically reshapes the synergistic ecology of kindergartens by constructing a shared vision (e.g., formulating a synergistic development plan for the home and community), implementing intellectual stimulation (e.g., organizing cross-disciplinary teaching and research activities), and providing personalized care (e.g., supporting teachers’ professional growth). Although this type of organizational change does not directly involve teacher-child interactions, it provides institutional safeguards and cultural soil for young children’s social–emotional development, which is consistent with the assumption of transformational leadership theory that “transformational leadership influences distal outcomes through organizational climate” (Xinping, 2008).

Additionally, the theory of transformational leadership is consistent with the assumption that transformational leadership influences distal outcomes through organizational climate. Simultaneously, teachers’ socioemotional competence serves as the main vehicle for the transformation of collaborative resources into educational practice, and its mechanism of action fits the “triadic interaction model” of social cognitive theory. Teachers’ self-efficacy is not only enhanced through the construction of social support networks (e.g., parent participation in curriculum design) but also through the creation of practice areas (e.g., community volunteering), which facilitates the embodied acquisition of teachers’ affective guidance strategies. The enhancement of this ability can further nourish children’s social development through the “recent processes” of emotional response and conflict mediation in daily interactions between teachers and children (Liu, 2024). The study results have two implications for practice: first, the strategic role of the director in the construction of the collaborative mechanism should be strengthened, and his/her capacity for resource integration and organizational change should be reinforced through leadership training; second, the professional development of teachers’ socioemotional competence should be emphasized, and the in-depth coupling of home-school collaborative practice and teachers’ training should be established. Future research could further explore the synergistic mechanism of the two paths, particularly how directors’ leadership can amplify the synergistic effect of nurturing by empowering the teacher community.

### 5.4 Chain mediating roles of preschool preschoolers’ socioemotional competence and principals’ transformational leadership

This study further elucidates a chained mediating pathway: “home-kindergarten-community collaboration → principals’ transformational leadership → teachers’ socioemotional competence → preschoolers’ socioemotional competence.” The findings indicate that the level of home-kindergarten-community collaboration indirectly influences the development of preschoolers’ socioemotional competence through a series of sequential mediations, thereby substantiating the complex interactions among “elements, structure, and levels” as proposed in ecological systems theory. Specifically, the outer system (principals’ transformational leadership) exerts its influence on the microsystem (young children’s development) through the mesosystem (teachers’ practices). Within this framework, home-kindergarten-community collaboration acts as the core driving force for the mesosystem by encouraging principals to reshape the organizational environment of kindergartens through transformational leadership (e.g., vision building and resource integration). However, the impact of this leadership style on preschoolers’ socioemotional competence is fully mediated by teachers’ socioemotional competence. This mediation occurs because the development of young children primarily relies on the direct “proximal process,” namely, high-quality teacher-child interactions. This discovery substantiates the following: the core proposition in ecosystem theory that “cross-level effects must be transmitted through direct interacting agents”; the critical role of kindergarten principals and teachers in vicarious reinforcement and modeling as described in social learning theory. Therefore, kindergarten principals should fully demonstrate their exemplary leadership skills in establishing a home-kindergarten-community collaboration mechanism by showcasing competencies such as strategic direction-setting, teaching quality enhancement, human resource development, and organizational restructuring. This will provide strong support for preschool teachers, enabling them to enhance their abilities in self-awareness, self-regulation, social cognition, interpersonal communication, and responsible decision-making, while subtly cultivating a supportive material and emotional environment conducive to the development of children’s social and emotional competencies. Given that this study used a cross-sectional design, future research could consider adopting a longitudinal follow-up approach to more clearly delineate the relationships among the various variables (Han et al., 2024).

## 6 Conclusion

### 6.1 Implications for principals’ transformational leadership

The study findings indicate that principals’ transformational leadership can influence preschoolers’ socioemotional competence development through a home-kindergarten-community collaboration mechanism, indirectly promoting children’s growth by enhancing teachers’ socioemotional competence. Consequently, kindergarten principals’ leadership roles should be fully leveraged within the framework of collaborative education. First, principals should establish clear, context-specific goals and systematically design and implement strategies to improve teachers’ socioemotional competence. Simultaneously, they should collaborate with local universities to introduce the “dual mentor system,” providing professional guidance and support for teachers via this system while formulating a tiered development plan. Second, they should reshape the organizational ecology of kindergartens by transcending traditional management models, integrating external resources into kindergarten operations, and embedding the cultivation of teachers’ socioemotional competence as a core component of the daily teaching and research system.

### 6.2 Implications for preschool teachers’ socioemotional competence

The current study indicates that within the home-kindergarten-community collaboration framework, preschool teachers’ socioemotional competence serves as a critical mediating factor that significantly influences the development of preschoolers’ socioemotional competence. In other words, teachers’ socioemotional competence subtly but profoundly shapes children’s corresponding skills. Hence, kindergarten teachers can be encouraged to document typical multimodal cases of children’s daily kindergarten life (e.g., language, behavior, activities) for a comprehensive evaluation of preschoolers’ socioemotional performance (Chunyan and Dandan, 2023). Moreover, institutionalized approaches can support teachers in systematically analyzing and reflecting on these cases during school-based teaching and research activities. Collaboration with university teacher educators can help to clarify the significance and construct the meaning of relevant cases, thereby enhancing preschool teachers’ professional role awareness and their ability to interpret children’s behaviors from a socioemotional perspective. Finally, guiding teachers to integrate these insights into classroom teaching practices can enable them to serve as role models, creating vicarious reinforcement for children while providing emotional connections and support to foster preschoolers’ socioemotional competence.

### 6.3 Implications for home-kindergarten-community collaborative co-education network

The study findings indicate that collaboration between homes, kindergartens, and communities not only directly fosters preschoolers’ socioemotional competence but also indirectly facilitates their development through enhanced principals’ transformational leadership and improved teachers’ socioemotional competence. These results highlight the critical role of such collaboration in nurturing preschoolers’ socioemotional competence. Therefore, strengthening home-kindergarten-community collaboration and establishing diverse support systems are effective strategies for promoting preschoolers’ socioemotional development.

1. For home-kindergarten cooperation: First, kindergartens should consider the unique needs and characteristics of different families when making decisions and organizing activities. Strategically utilizing family resources can help kindergartens actively involve parents in their educational and managerial processes. Furthermore, kindergartens should regularly reflect on and adjust participation content, formats, and plans through collaborative discussions with parents to ensure that their opinions and suggestions are genuinely valued. This approach continuously enhances the effectiveness and depth of parental involvement. Second, kindergartens should systematically conduct regular investigations into the current state of and needs for family education. Based on these findings, kindergartens can design and implement highly targeted family education guidance activities tailored to meet parents’ specific needs. Additionally, it is essential to scientifically evaluate the efficacy of these activities and refine the guidance plans based on the results, thereby ensuring the scientific rigor and high quality of family education guidance.
2. For homes-kindergartens-communities collaboration: First, kindergartens should make a comprehensive inventory of the surrounding communities’ resources and categorize them. Next, they should develop a systematic, structured community resource database to assess the value and applicability of the available resources. Meanwhile, partnerships with primary schools should be strengthened by designing activities for the transition from kindergartens to primary schools. These activities facilitate children’s smooth adaptation and leverage the supportive role of community resources in early childhood education. Second, the kindergarten’s professional capacity to serve the community can be enhanced through dynamic investigations and analyses of community needs. Additionally, the content and format of professional services can be adjusted to ensure their effectiveness. These improvements will significantly enhance service quality and foster positive interactions and deeper collaborations between kindergartens and the community.

However, this study is not without its limitations, which merit further refinement in subsequent research. First, the cross-sectional research design used in this study did not allow for causal inference; a longitudinal design or experimental research methodology could be considered for exploring the causal relationship between home-society collaboration and children’s socioemotional competence. Such exploration could be achieved by employing an aggregated crossover design, a multilayered linear model, or manipulation of the independent variable and mediator variables. Second, the study employed a balanced sample size in Guangdong and Yunnan Provinces, encompassing a range of scenarios including urban and rural areas. However, the model’s construction and mediation effect analysis did not incorporate demographic variables, which may have led to a reduction in the model’s explanatory power. Due to the constraints imposed by the scope of this paper, a thorough exploration of this issue was not feasible. Future research endeavors could involve a more comprehensive comparison of the disparities among different regions and sample types, along with a thorough analysis of the underlying causes of these variations. The establishment of a robust ecosystem for early childhood development education is predicated on the synergistic cooperation among families, kindergartens, and communities. This collaborative effort is instrumental in fostering a scientific basis for the effective execution of home and family functions.

## Data Availability

The raw data supporting the conclusions of this article will be made available by the authors, without undue reservation.
